# Isothiocyanate-Rich Essential Oil of *Morisonia flexuosa* L. Exhibits Anxiolytic-like Effects That May Involve Serotonergic Pathways in Zebrafish

**DOI:** 10.3390/plants15121812

**Published:** 2026-06-12

**Authors:** Fázia Fernandes Galvão Rodrigues, Natalia Kelly Gomes de Carvalho, Geane Gabriele de Oliveira Souza, Hélcio Silva dos Santos, Irwin Rose Alencar de Menezes, Amanda Maria Barros Alves, Jane Eire Silva Alencar de Menezes, Fabiola Fernandes Galvão Rodrigues, José Galberto Martins da Costa

**Affiliations:** Natural Products Research Laboratory, Department of Biological Chemistry, Universidade Regional do Cariri, Rua Coronel Antônio Luíz, 1161—Pimenta, Crato 63105-010, CE, Brazil; fazia.galvao@urca.br (F.F.G.R.); nataliakellygc@gmail.com (N.K.G.d.C.); geane.souza@urca.br (G.G.d.O.S.); helciodossantos@gmail.com (H.S.d.S.); irwin.alencar@urca.br (I.R.A.d.M.); amanda.barros@aluno.uece.br (A.M.B.A.); jane.menezes@uece.br (J.E.S.A.d.M.); fabiolafer@gmail.com (F.F.G.R.)

**Keywords:** anxiolytic-like activity, isothiocyanates, serotonergic modulation, zebrafish model, essential oil, principal component analysis

## Abstract

Anxiety disorders are characterized by dysregulation of monoaminergic signaling and remain a significant therapeutic challenge due to limitations associated with current pharmacological treatments. In this context, the essential oil of *Morisonia flexuosa* (Capparaceae) seeds was chemically characterized and evaluated for anxiolytic-like activity in adult zebrafish. Chemical profiling by GC–MS and GC–FID revealed a predominance of isothiocyanates, particularly butyl isothiocyanate (42.60%) and isobutyl isothiocyanate (42.28%). Acute toxicity assessment demonstrated no lethality at the tested doses. Behavioral analyses showed a significant increase in light preference in the light/dark paradigm, with moderate locomotor reduction insufficient to account for the behavioral shift solely by sedation. Pharmacological antagonism assays indicated that the anxiolytic-like effect was predominantly mediated by 5-HT1 and 5-HT2A/2C receptors. Chemometric analyses (PCA, HCA, and heatmap) revealed statistical association between compound abundance and behavioral endpoints, supporting the contribution of major isothiocyanates within the tested model. Notably, the strongest behavioral response was observed at the lowest concentration, suggesting an ideal effective concentration range. Collectively, these findings provide the first evidence that an isothiocyanate-rich essential oil from *M. flexuosa* exerts serotonergic-involved anxiolytic-like effects in zebrafish and supports further mechanistic investigation of its neuropharmacological potential.

## 1. Introduction

Anxiety disorders encompass a heterogeneous group of psychiatric conditions, including generalized anxiety disorder, panic disorder, obsessive–compulsive disorder, phobias, and post-traumatic stress disorder, and constitute one of the leading causes of global disability. According to epidemiological estimates, anxiety-related disorders affect hundreds of millions of individuals worldwide and are associated with significant socioeconomic burden, impaired quality of life, and increased comorbidity with depressive and neuroinflammatory conditions. Neurobiologically, anxiety disorders are characterized by dysregulation of monoaminergic systems (serotonergic, noradrenergic, and dopaminergic pathways), hyperactivity of the hypothalamic–pituitary–adrenal (HPA) axis, and alterations in corticolimbic circuits involving the amygdala, hippocampus, and prefrontal cortex [1,2].

Pharmacological treatment traditionally relies on benzodiazepines and selective serotonin reuptake inhibitors (SSRIs). Benzodiazepines exert their anxiolytic action via positive allosteric modulation of GABA-A receptors; however, their clinical use is limited by sedation, psychomotor impairment, tolerance, dependence, and withdrawal phenomena [3]. SSRIs, although safer in long-term use, often require prolonged administration and may initially exacerbate anxiety symptoms due to acute serotonergic overstimulation. Moreover, emerging evidence indicates that a subset of patients exhibits treatment-resistant anxiety, highlighting the need for multi-target therapeutic approaches [4].

In this context, natural products have gained increasing attention as potential sources of novel anxiolytic agents. Plant-derived compounds, particularly those found in aromatic species, have demonstrated neuromodulatory properties in preclinical models. Essential oils are complex mixtures of volatile secondary metabolites, including terpenes, phenylpropanoids, and sulfur-containing compounds, that can cross biological membranes due to their lipophilic nature. The multicomponent character of essential oils allows simultaneous interaction with multiple molecular targets, including gabaergic, serotonergic, glutamatergic, and endocannabinoid systems, which may result in synergistic or additive neuropharmacological effects [5].

Experimental evidence has demonstrated anxiolytic-like activity for several essential oils and isolated phytochemicals, with mechanisms involving modulation of 5-HT1A receptors, inhibition of monoamine oxidase, and attenuation of neuroinflammatory pathways. Increasing attention has also been given to the role of oxidative stress and neuroinflammation in the pathophysiology of anxiety disorders, where redox imbalance may alter serotonin synthesis and receptor expression [6]. Consequently, compounds capable of activating endogenous antioxidant pathways, such as the Nrf2 signaling cascade, have emerged as promising candidates for neuropsychiatric modulation [7].

In parallel, zebrafish (*Danio rerio*) has become an established vertebrate model for neurobehavioral pharmacology. Zebrafish share high genetic homology with humans and possess conserved monoaminergic systems, including serotonin receptors homologous to mammalian 5-HT subtypes [8,9]. Behavioral paradigms such as the light/dark preference test and open field test have been validated for assessing anxiety-like behavior and pharmacological responses [10]. Additionally, zebrafish offer the advantage of high-throughput screening and translational relevance in studies investigating neurotoxicity, oxidative stress, and psychotropic effects [2].

*Morisonia flexuosa* (syn. *Cynophalla flexuosa* L.), popularly known as “feijão-bravo,” belongs to the Capparaceae family and is widely distributed in the Brazilian Caatinga biome, particularly in semi-arid regions [11,12]. Although phytochemically underexplored, previous investigations have reported the presence of glucosinolates in different plant parts. Upon enzymatic hydrolysis by myrosinase, glucosinolates yield isothiocyanates, electrophilic organosulfur compounds with recognized pharmacological relevance [12,13].

Isothiocyanates (ITCs) possess well-documented anti-inflammatory, antimicrobial, and anticancer activities [6,14]. These compounds, naturally abundant in cruciferous vegetables such as broccoli and cauliflower, exert their biological effects primarily through covalent modification of cysteine residues in regulatory proteins, leading to activation of the Keap1–Nrf2 pathway and induction of antioxidant and cytoprotective genes [7,15]. Recent studies suggest that nrf2 activation may indirectly modulate monoaminergic neurotransmission by reducing neuroinflammatory signaling and mitochondrial dysfunction, factors that contribute to anxiety-like phenotypes [6].

Despite extensive evidence supporting the chemopreventive and antioxidant properties of ITCs, their potential role in modulating anxiety-related behavior remains largely unexplored. Therefore, investigating essential oils rich in isothiocyanates may contribute to identifying novel neuroactive compounds capable of interacting with serotonergic pathways and redox-sensitive mechanisms.

Accordingly, the present study aimed to characterize the chemical profile of the essential oil obtained from *M. flexuosa* seeds through chromatographic analysis and to evaluate its acute toxicity and anxiolytic-like activity using validated zebrafish behavioral assays. By integrating phytochemical characterization, behavioral pharmacology, and neurochemical modulation studies, this work seeks to advance the understanding of isothiocyanate-rich essential oils as multi-target candidates for anxiety management.

## 2. Results

### 2.1. Chemical Composition

**Figure 1:**
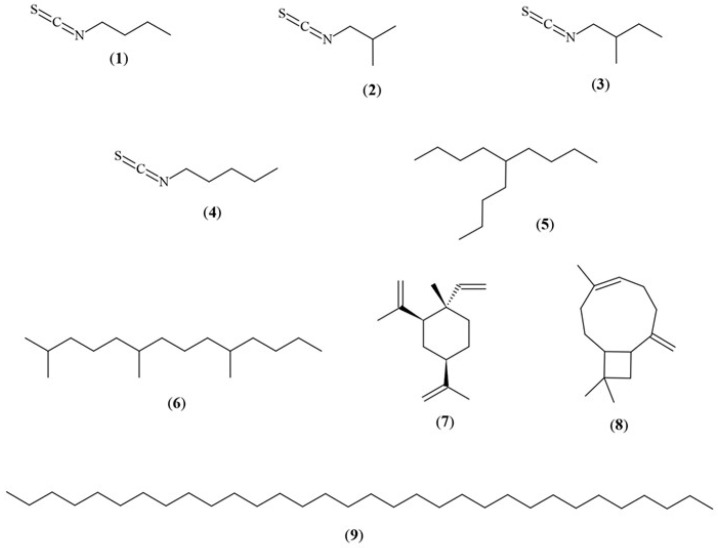
Chemical components identified in the essential oil of *M. flexuosa* seeds. (1) Butyl isothiocyanate, (2) Isobutyl isothiocyanate, (3) 2-Methylbutyl isothiocyanate, (4) Pentyl isothiocyanate, (5) 5-Butylnonane, (6) 2,6,10 Trimethyltetradecane, (7) (−)-β-Elemene, (8) *trans*-Caryophyllene, (9) Dotriacontane.

The compounds identified in the essential oil of *M. flexuosa* seeds (EOMF) by GC–MS and confirmed by GC–FID accounted for 94.17% of the total chemical composition (Table 1), of which 87.80% belonged to the isothiocyanate class. The major constituents were the isomers butyl isothiocyanate (42.60%) and isobutyl isothiocyanate (42.28%) (Figure 1), which together accounted for 84.88% of the identified components (Table 1).

Isothiocyanates are organosulfur compounds characterized by the functional group R–N=C=S, whose electrophilic nature arises from the electronegativity of nitrogen and sulfur atoms, rendering the central carbon susceptible to nucleophilic reactions and conferring biological activity [16]. Their formation and stability are influenced by factors such as pH, temperature, ferrous ion availability, and the presence of proteins including the epithiospecifier protein, and due to their thermolabile character, careful extraction is required [15].

The isothiocyanates identified in the present study, compounds **1**–**4**, exhibit relatively low molecular weight, which contributes to their volatility and to the characteristic aroma of the plant, and possess variable aliphatic R-chains that may include primary and secondary carbons (non-methylated) or primary, secondary, and tertiary carbons (methylated). Notably, the shorter-chain derivatives, butyl isothiocyanate and isobutyl isothiocyanate, were the predominant constituents of the essential oil, suggesting that structural features such as chain length and branching may influence both volatility and relative abundance within the phytochemical profile [17].

Compounds **5**, **6**, and **9** are aliphatic hydrocarbons containing 13, 16, and 32 carbon atoms, respectively. Their structures comprise primary, secondary, and/or tertiary carbons, classifying them as saturated and poorly branched compounds (Figure 1).

The EOMF content was also marked by the presence of monocyclic 7 and bicyclic 8 sesquiterpenes. Despite their greater structural complexity compared with the other compounds, both are composed exclusively of carbon and hydrogen atoms, exhibiting multiple branches and unsaturations (Figure 1).

Isothiocyanate compounds have also been identified in different parts of the plant. Carvalho et al. (2021) [13] confirmed the presence of ITCs and nitriles (39.4%) in the essential oil of the leaves and isopropyl isothiocyanate (25.9%) in the essential oil of the bark of *M. flexuosa*.

### 2.2. Safety Profile and Behavioral Specificity

**Figure 2:**
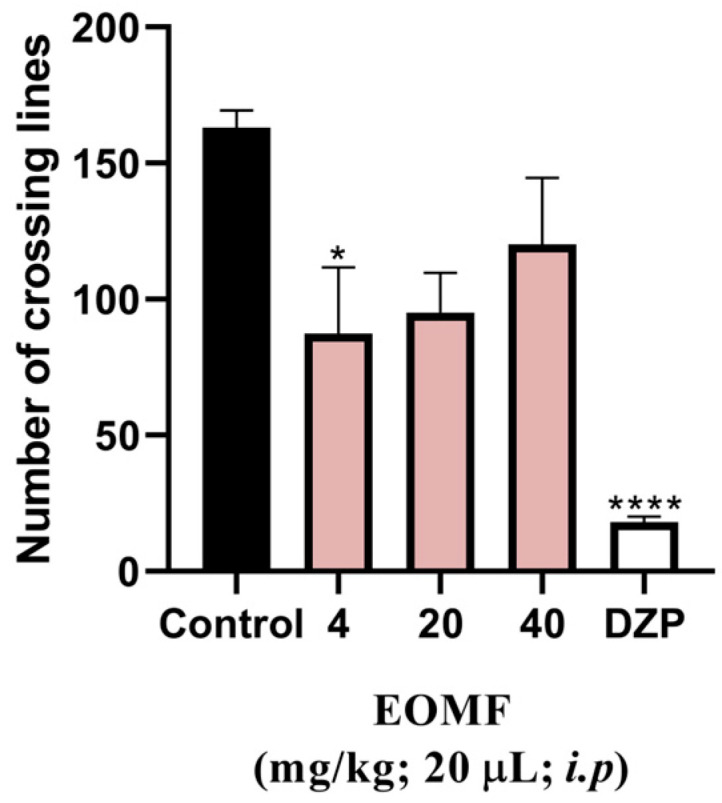
Effect of the essential oil of *M. flexuosa* seeds (EOMF) on the locomotor behavior of adult zebrafish (*Danio rerio*) in the Open Field Test (0–5 min). Control (3% DMSO; 20 µL; i.p.) —untreated animals. DZP—diazepam (4 mg/kg; 20 µL; i.p.). Values represent mean ± standard error of the mean (SEM) for six animals per group; one-way ANOVA followed by Tukey’s test. (* *p* < 0.05; **** *p* < 0.0001 vs. Control).

The essential oil EOMF did not induce mortality or observable anatomical changes in adult zebrafish during the 96-h exposure period, with an estimated LD_50_ > 40 mg/kg. No statistically significant mortality was recorded (*p* > 0.05), indicating low acute systemic toxicity under the tested conditions. Regarding animal locomotion, an alteration occurred only at the 4 mg/kg dose, as the animals showed reduced locomotor activity compared to the control group (Figure 2). However, at the higher doses analyzed (20 and 40 mg/kg), there was no reduction in locomotor activity (* *p* < 0.05; **** *p* < 0.0001 vs. Control).

### 2.3. Anxiolytic-like Activity and Dose–Response Interpretation

**Figure 3:**
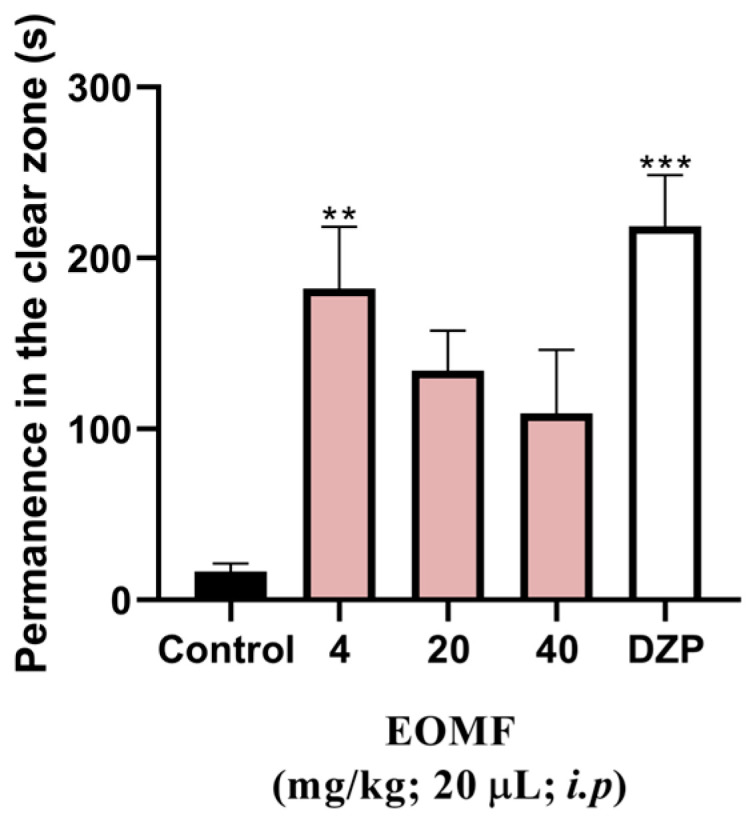
Effect of essential oil of *M. flexuosa* seeds (EOMF) on the anxiolytic effect of adult zebrafish in the light/dark test in seconds (s). Control (DMSO 3%; 20 µL; i.p.)—untreated animals. DZP—diazepam (4 mg/kg; 20 µL; i.p.). Values represent the mean ± standard error of the mean for *n* = 6; ANOVA followed by Tukey’s test. (** *p* < 0.01; *** *p* < 0.001).

The light/dark preference test was employed to evaluate anxiety-like behavior in the animals. As shown in Figure 3, treatment with EOMF at a dose of 4 mg/kg significantly increased the time spent in the illuminated compartment compared with the control group (** *p* < 0.01; *** *p* < 0.001). These findings indicate a pronounced anxiolytic-like effect, comparable to that produced by diazepam (4 mg/kg), which was used as the standard anxiolytic drug.

### 2.4. Neuropharmacological Mechanisms: GABAergic and Serotonergic Modulation

**Figure 4:**
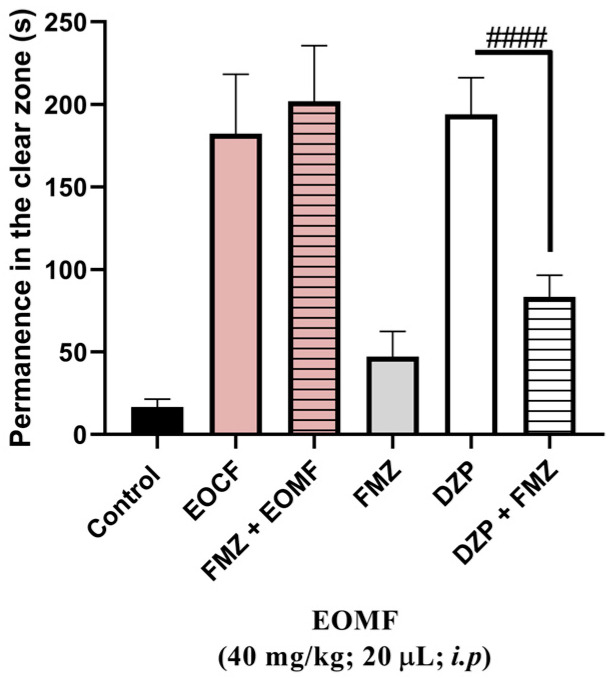
Effect of the essential oil of *M. flexuosa* seeds (EOMF) on GABAergic neuromodulation in zebrafish in the light/dark test in seconds (s). Control (3% DMSO; 20 µL; i.p.)—untreated animals. DZP—diazepam (4 mg/kg; 20 µL; i.p.). FMZ—flumazenil (4 mg/kg; 20 µL; i.p.). Values represent mean ± standard error of the mean (SEM) for *n* = 6; one-way ANOVA followed by Tukey’s test. (#### *p* < 0.0001).

To investigate whether the anxiolytic-like effect of EOMF involved classical GABAergic pathways, the animals were pretreated with flumazenil, a selective antagonist of the benzodiazepine-binding site on GABA-A receptors. As shown in Figure 4, flumazenil failed to reverse the anxiolytic-like effect induced by EOMF; instead, a 17.60% increase in the time spent in the light zone was observed. These findings suggest that the anxiolytic-like activity of EOMF is not mediated through the benzodiazepine-binding site of GABA-A receptors (#### *p* < 0.0001).

**Figure 5:**
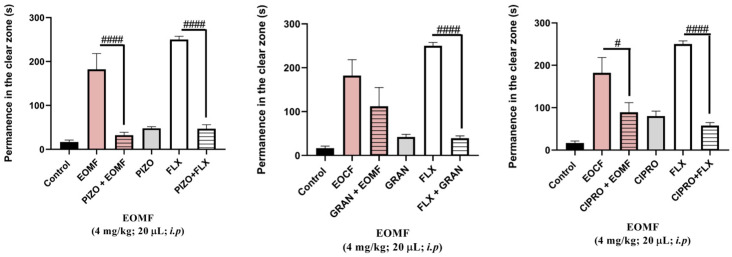
Effect of the essential oil of *M. flexuosa* seeds (EOMF) on serotonergic neuromodulation in adult zebrafish in the light/dark test in seconds (s). Control (3% DMSO; 20 µL; i.p.)—untreated animals. Fluoxetine (FLX; 0.05 mg/kg; 20 µL; i.p.). GRAN—granisetron (20 mg/kg; oral route). PIZO—pizotifen (32 mg/kg; i.p.). CIPRO—cyproheptadine (32 mg/kg; i.p.). Values represent mean ± standard error of the mean (SEM) for *n* = 6; one-way ANOVA followed by Tukey’s test. (# *p* < 0.1; #### *p* < 0.0001).

To further clarify the mechanism, serotonergic antagonists were administered. As demonstrated in Figure 5, co-administration of cyproheptadine, pizotifen, and granisetron significantly reduced the anxiolytic-like effect of EOMF, with decreases of 66.60%, 84.40%, and 41.70%, respectively.

Pharmacological antagonism experiments further demonstrated that the anxiolytic-like effect was not dependent on benzodiazepine-site GABA-A receptor modulation in (Figure 4) but was significantly attenuated by serotonergic antagonists (Figure 5), supporting predominant involvement of 5-HT1 and 5-HT2A/2C receptors. Collectively, these findings suggest that EOMF acts through a serotonergic-centered mechanism, potentially influenced by redox-sensitive signaling pathways, within an optimal effective concentration range.

### 2.5. Chemometric Analysis

**Figure 6:**
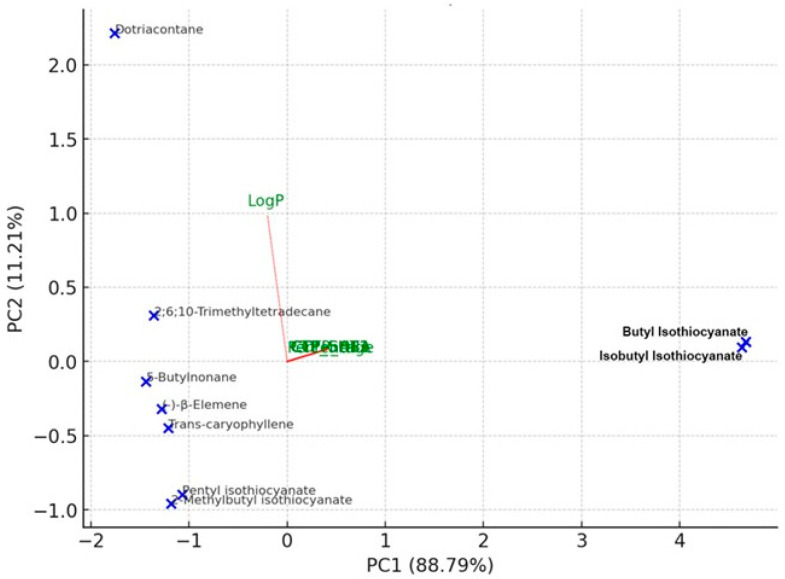
Principal Component Analysis (PCA) of the essential oil of *M. flexuosa* seeds (EOMF).

Principal Component Analysis (PCA) revealed that PC1 explained 88.79% of total variance, while PC2 accounted for 11.21% (Figure 6). PC1 was strongly associated with compound percentage and behavioral variables (5-HT1, 5-HT2, 5-HT3, and open field), which appeared to align positively along the same axis. PC2 was primarily associated with LogP values, indicating that lipophilicity contributed to secondary variance. However, the high percentage of variance explained by PC1 indicates that compound abundance was the dominant structuring factor in the dataset. Importantly, because the biological activity values used in the matrix were proportionally distributed according to compound abundance rather than experimentally determined for each isolated constituent, the observed associations should be interpreted cautiously. Thus, the PCA primarily reflects statistical covariance driven by the weighted model structure and does not demonstrate direct compound-specific pharmacological activity or mechanistic relationships.

**Figure 7:**
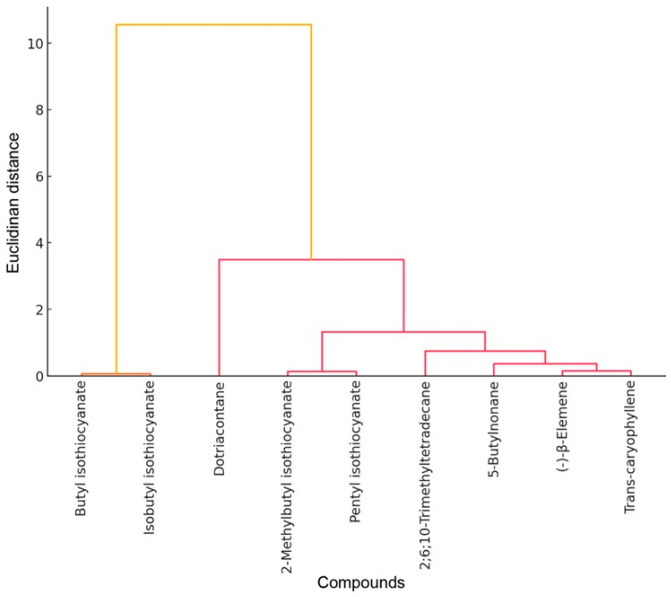
Cluster analysis of the essential oil of *M. flexuosa* seeds (EOMF).

Hierarchical Cluster Analysis (HCA) grouped compounds into four clusters, primarily based on chemical similarity and proportional contribution (Figure 7). Cluster 1 included butyl and isobutyl isothiocyanate, reflecting both structural similarity and their predominance in the oil composition. Clusters 2–4 separated minor constituents according to physicochemical characteristics, particularly lipophilicity and structural differences. Nevertheless, because the chemometric matrix incorporated replicated global bioactivity values weighted by relative abundance, the clustering pattern is strongly influenced by compositional dominance rather than independent experimental evidence of biological similarity. Therefore, these groupings should be considered exploratory statistical associations useful for hypothesis generation, but not confirmatory evidence of specific biological interactions or synergistic mechanisms.

However, because bioactivity values were replicated across compounds in the model, clustering partially reflects relative abundance rather than true biological independence. This reinforces the idea that the chemometric grouping here should be interpreted as exploratory rather than confirmatory.

**Figure 8:**
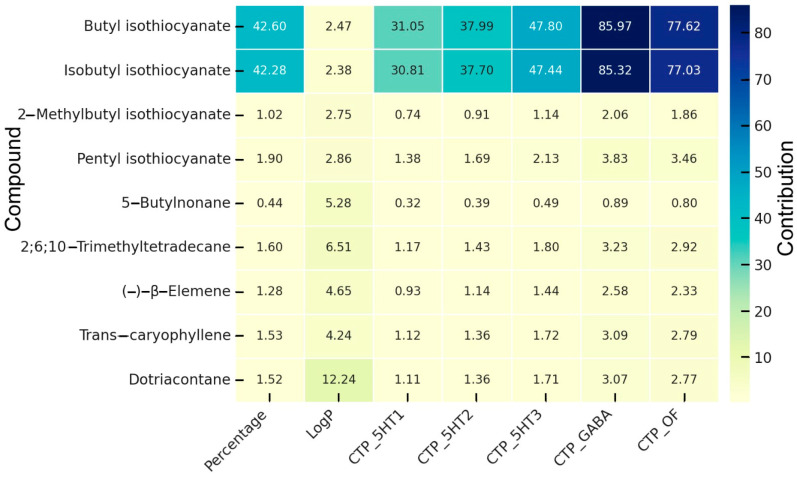
Correlation between concentration and bioactivity of the chemical components of EOMF.

The heatmap in Figure 8 indicated a strong positive correlation between compound percentage and behavioral endpoints. This outcome is expected because the weighted model assumes proportional contribution of each compound to the overall bioactivity profile. Consequently, the observed correlations predominantly reflect mathematical dependence within the constructed dataset rather than experimentally validated causal relationships [18]. For this reason, the correlation patterns should not be interpreted as direct evidence that individual constituents are solely responsible for the observed anxiolytic-like effects. Additional studies using isolated compounds, fractionation strategies, and targeted pharmacological assays are required to validate the biological relevance of these associations. Therefore, correlation does not necessarily imply causation; rather, it reflects mathematical dependence within the constructed matrix [18].

**Figure 9:**
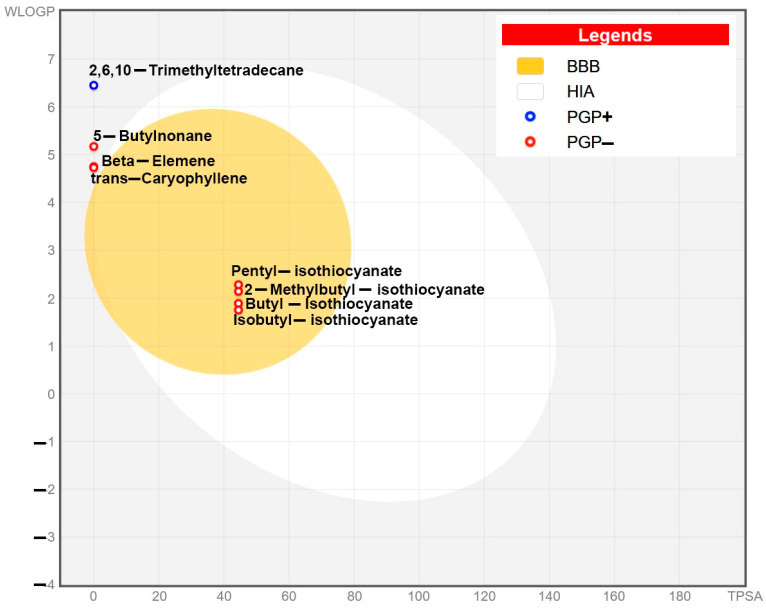
Bioavailability potential of the essential oil of *Morisonia flexuosa* (EOMF).

The BOILED-Egg model predicted gastrointestinal absorption and blood–brain barrier (BBB) penetration based on physicochemical properties in Figure 9. Compounds located within the white region indicate high predicted gastrointestinal absorption, whereas those in the yellow region (yolk) suggest high probability of BBB permeation.

EOMF exhibited a favorable acute safety profile in adult zebrafish and produced a significant anxiolytic-like effect at non-toxic doses. Behavioral data demonstrated increased light preference in the light/dark paradigm, with a moderate reduction in locomotor activity insufficient to explain the effect solely as sedation. Pharmacological antagonism assays indicated that the anxiolytic-like response was independent of benzodiazepine-site GABA-A receptor modulation and predominantly may involve through serotonergic pathways, particularly involving 5-HT1 and 5-HT2A/2C receptors.

Chemometric analyses revealed a statistical association between major isothiocyanates, especially butyl and isobutyl isothiocyanates, and behavioral endpoints, which were largely influenced by their relative abundance and physicochemical properties. However, because the multivariate models were constructed using weighted replication of global bioactivity values across compounds, the resulting associations mainly reflect abundance-driven statistical relationships rather than experimentally confirmed compound-specific activities. Therefore, these analyses should be interpreted as exploratory tools for identifying potential contributors to the observed pharmacological profile and generating hypotheses for future investigation, rather than as definitive mechanistic evidence.

Overall, the integration of phytochemical characterization, behavioral pharmacology, neuromodulatory assays, and in silico predictions supports the neuroactive potential of EOMF and suggests serotonergic modulation as a relevant pathway involved in the anxiolytic-like effect. Nonetheless, direct molecular targets and the specific contribution of individual constituents remain to be experimentally established through bioactivity-guided isolation and mechanistic studies.

## 3. Discussion

According to OECD guidelines [19], the absence of lethality within this range suggests that EOMF does not produce severe systemic toxicity. Zebrafish represent a validated vertebrate model for toxicological screening due to conserved xenobiotic metabolism pathways. This safety profile is corroborated by previous toxicological assessments. Rudzińska-Radecka et al. [20] demonstrated that a sulforaphane phosphonate analog (P-ITC; 1–3 µM) was not toxic to zebrafish embryos at different developmental stages (6 and 48 hpf). Similarly, Mendes et al. [21] reported that the essential oil of *Marlierea eugeniopsoides* was non-toxic and well tolerated in adult zebrafish at all doses tested. Taken together, these reports corroborate that plant-derived essential oils, including isothiocyanate-rich EOMF, exhibit low acute toxicity in the zebrafish model [8,9].

The lack of acute toxicity is particularly relevant considering the electrophilic nature of isothiocyanates, which may interact with cellular thiol groups at high concentrations [15], but at moderate doses can activate adaptive cytoprotective pathways such as Nrf2 [7].

To determine whether behavioral changes were secondary to nonspecific motor suppression, locomotor activity was assessed in the open-field test.

In zebrafish anxiety paradigms, true anxiolytics may reduce exploratory hyperactivity without abolishing locomotor capacity [1,10]. The data presented in Figure 2 suggest that EOMF does not produce profound motor impairment, supporting the interpretation that subsequent anxiolytic-like effects are not merely a consequence of sedation.

Regarding chemical composition, β-caryophyllene, although present in lower proportions, has documented anxiolytic activity mediated via CB_2_ and GABAergic modulation [22,23]. Conversely, β-elemene has been associated with anxiety-like behavior in murine models [24].

This suggests that the final behavioral outcome may result from a balance between multiple bioactive constituents, rather than a single dominant compound.

Importantly, higher concentrations did not produce greater anxiolytic-like responses, indicating a non-linear dose–response pattern. The inverted U-shaped profile observed in Figure 3 is consistent with hormetic pharmacological responses, commonly reported for phytochemicals that modulate redox-sensitive or neurotransmitter systems. This inverted U-shaped pattern is consistent with hormetic responses described for phytochemicals that activate the Nrf2 pathway, such as sulforaphane, where moderate doses induce cytoprotection while higher doses may overwhelm antioxidant capacity [4,25].

Isothiocyanates activate the Nrf2 pathway [7], which modulates oxidative stress and inflammatory mediators. Oxidative imbalance has been shown to alter serotonergic neurotransmission and receptor expression [6].

This differentiates EOMF from benzodiazepines, which exert anxiolytic effects through positive allosteric modulation of GABA-A receptors [3]. Thus, the behavioral shift toward light preference observed in Figure 3 may reflect indirect serotonergic modulation through redox-dependent mechanisms rather than a direct receptor-agonist effect. The absence of dose-dependent enhancement suggests that major isothiocyanates alone may not fully account for the behavioral response. This supports a multi-target pharmacological model within an optimal effective concentration range.

The strongest attenuation occurred with pizotifen, a 5-HT1 and 5-HT2A/2C antagonist, indicating that serotonergic modulation is central to the observed behavioral effect. The serotonergic system plays a pivotal role in anxiety regulation, with 5-HT1A receptor activation associated with anxiolysis and 5-HT2 receptor modulation influencing emotional reactivity [2,10].

To further clarify the mechanism, serotonergic antagonists were administered. As demonstrated in Figure 5, co-administration of cyproheptadine, pizotifen, and granisetron significantly reduced the anxiolytic-like effect of EOMF, with decreases of 66.60%, 84.40%, and 41.70%, respectively. Our findings align with those of Benneh et al. [26], who demonstrated that the anxiolytic-like effect of *Maerua angolensis* extract was similarly attenuated by pizotifen and cyproheptadine, reinforcing the conserved role of 5-HT1 and 5-HT2 receptors in zebrafish anxiety models.

The data presented in Figure 5 provide strong evidence that the 5-HT1 and 5-HT2A/2C serotonergic receptors are involved in the observed effect. Considering the electrophilic properties of isothiocyanates and their ability to activate cytoprotective pathways [7], it is plausible that EOMF enhances serotonergic signaling indirectly via redox-sensitive modulation of neuronal homeostasis [6]. Therefore, the combined pharmacological data in Figure 4 and Figure 5 indicate that EOMF does not act as a GABAergic benzodiazepine-like agent but rather as a multi-target modulator with prominent serotonergic involvement.

Taken together, the behavioral and pharmacological findings allow for the construction of a mechanistic model, which is corroborated by the data presented in Figure 2, Figure 3, Figure 4 and Figure 5. EOMF exhibited a favorable acute safety profile within the tested range, with no evidence of systemic toxicity. Although a moderate reduction in locomotor activity was observed in Figure 2, the magnitude of this effect was insufficient to account for the robust anxiolytic-like response detected in the light/dark paradigm in Figure 3, indicating that behavioral modulation was not merely a consequence of sedation.

Compared with other plant-derived essential oils with documented anxiolytic activity, such as those from *Lippia alba* and *Ocimum basilicum*, which act primarily via GABAergic modulation, EOMF appears to engage a distinct serotonergic pathway. This mechanistic divergence may be advantageous for patients with benzodiazepine-resistant anxiety or those seeking alternatives with reduced sedative liability [2,3,10].

This indicates that the PCA structure is largely driven by relative concentration rather than intrinsic pharmacodynamic properties, a common limitation in multivariate analysis of essential oils [18]. Compounds such as butyl and isobutyl isothiocyanate appeared isolated along PC1, reflecting their high relative abundance.

It is important to emphasize that PCA loadings do not demonstrate direct receptor interaction or molecular target engagement; rather, they indicate statistical co-variation between variables [26]. Thus, the positioning of these compounds near serotonergic variables does not establish direct 5-HT receptor inhibition but suggests statistical association within the constructed dataset.

While butyl and isobutyl isothiocyanate exhibited the highest theoretical contributions (>30%), this reflects concentration dominance. Nevertheless, concentration remains a relevant pharmacological determinant in essential oil research, as major constituents frequently contribute substantially to biological effects [27].

The previous interpretation that yolk positioning indicates low intestinal absorption is incorrect. Rather, compounds may display both high GI absorption and BBB permeability depending on their location within the model [27]. Extremely lipophilic compounds, such as dotriacontane (LogP > 10), fall outside the optimal absorption-permeability window, suggesting limited systemic bioavailability.

The chemometric results should be interpreted with caution. Although PCA in Figure 6, HCA in Figure 7, and the heatmap in Figure 8 indicate an association between compound abundance and behavioral variables, the high variance explained by PC1 (88.79%) suggests that relative concentration predominantly structured the model. In multivariate analyses of complex mixtures, major constituents often drive variance independently of intrinsic pharmacodynamic potency [26]. Likewise, the Weighted Theoretical Contribution approach reflects proportional abundance and does not account for receptor affinity or synergistic interactions, which are common in essential oils [28]. Therefore, while the chemometric findings align with the serotonergic modulation observed in Figure 5, they should be considered exploratory rather than confirmatory evidence of molecular targeting.

## 4. Materials and Methods

### 4.1. Plant Material

The collection of *M. flexuosa* seeds was carried out in April 2024 in the district of Betânia, municipality of Farias Brito, Ceará, Brazil (7°14′95″ S; 39°29′73″ W). Taxonomic identification was performed after processing the botanical material according to standard herbarium procedures, followed by deposition in the collection of the Dárdamo de Andrade Lima Herbarium (URCA), under registration number 17044, under the responsibility of Dr. Maria Arlene Pessoa Silva.

### 4.2. Essential Oil Extraction

The essential oil extracted from *M. flexuosa* seeds (EOMF), obtained from 500 g of plant material, was produced by hydrodistillation using a Clevenger-type apparatus for 2 h. After extraction, the oil was dried over anhydrous sodium sulfate (Na_2_SO_4_), yielding 0.14% (*w*/*w*), and stored under refrigeration at 4 °C until chemical analyses and bioassays were performed.

### 4.3. GC–MS and GC–FID Analysis

The chemical composition of the essential oil was determined by GC–MS (qualitative) and GC–FID (quantitative) using Shimadzu GC–MS QP2010 and GC-2010 Plus systems (Shimadzu Scientific Instruments Inc., Columbia, MD, USA), equipped with an SH-Rtx-5 fused silica capillary column (30 m × 0.25 mm i.d.; 0.25 μm). The software used for the analyses was Compound Comoser Database, (Accessed on 11 May 2026).

The oven temperature program was 80–180 °C (4 °C/min), followed by heating to 246 °C (6.6 °C/min) and to 280 °C (3.4 °C/min), with a final hold of 10 min, totaling 30 min of analysis. Helium was used as the carrier gas (1.5 mL/min). A 1 µL aliquot of a 5 ppm solution in dichloromethane was injected in split mode (1:100 for GC–MS and 1:15 for GC–FID), with injector temperature set at 220 °C. For GC–MS, electron impact ionization at 70 eV was applied, with a scan range of 40–350 *m*/*z*, interface temperature of 280 °C, and ion source temperature of 200 °C. For GC–FID, the detector temperature was set at 300 °C. Compound identification was based on comparison of mass spectra with databases, software NIST 08 [29] and linear retention indices calculated using *n*-alkanes (C8–C40), according to Van den Dool and Kratz (1963) [30].

### 4.4. Drugs and Reagents

The following substances were used: Diazepam (DZP, Neo Química^®^, Fortaleza, CE, Brazil), dimethyl sulfoxide (3% DMSO; Dynamic^®^, São Paulo, Brazil), flumazenil, fluoxetine (FLX, Neo Química^®^), cyproheptadine (Sigma-Aldrich, St. Louis, MO, USA), granisetron (Sigma-Aldrich), and pizotifen (Sigma-Aldrich).

### 4.5. Zebrafish

Wild-type adult zebrafish (*Danio rerio*) (90–120 days old; 0.4 ± 0.1 g; 3.5 ± 0.5 cm), of both sexes, were obtained from a local supplier (Fortaleza, CE, Brazil). Animals were maintained in glass aquaria (30 × 15 × 20 cm) with a capacity of 10 L (*n* = 3/L), containing chlorinated water (ProtecPlus^®^, Santa Catarina, Brazil) and aeration provided by submerged air filters, at 25 °C and pH 7.0, under a 14/10 h light/dark circadian cycle. Fish were fed ad libitum with commercial feed (Spirulina^®^, Santa Catarina, Brazil) until 24 h before the experiments. Prior to drug administration, animals were anesthetized in ice-cold water (4–5 °C). After the experiments, euthanasia was performed by immersion in ice-cold water (2–4 °C) for 1 min until opercular movement ceased. All procedures were approved by the Animal Ethics Committee of the State University of Ceará (CEUA-UECE; No. 04983945/2021) and conducted in accordance with ethical principles for animal experimentation.

### 4.6. General Protocol

Zebrafish of both sexes were randomly selected, anesthetized in ice-cold water (4–5 °C), transferred to a moist sponge, and treated intraperitoneally with 20 µL of the sample at different doses (4, 20, and 40 mg/kg), or diazepam (4 mg/kg), or 3% DMSO, or fluoxetine. It is worth noting that the tests were performed blindly to minimize external interference and all behavioral scoring and subsequent data analyses were performed by experimenters who were blind to the experimental conditions, in order to reduce potential observational bias and ensure the reliability of the results.

#### 4.6.1. Acute Toxicity (96 h)

Acute toxicity was assessed following a single intraperitoneal (i.p.) administration of EOMF at doses of 4, 20, and 40 mg/kg (20 µL; *n* = 6/group). The protocol was adapted from the acute fish toxicity test (OECD TG 203) [19], as detailed in a standardized procedure for injected substances. The injection technique followed the method described by D.C. Patricia et al. [31]. After injection, the animals were returned to their aquaria and observed for 96 h, with mortality recorded every 24 h. The water was renewed daily as part of routine husbandry to maintain optimal water quality, not as a route of exposure. No mortality was observed at any of the tested doses, and the estimated LD_50_ was >40 mg/kg, as determined by the Trimmed Spearman–Karber method with a 95% confidence interval.

#### 4.6.2. Locomotor Activity Assessment (Open Field Test)

**Figure 10:**
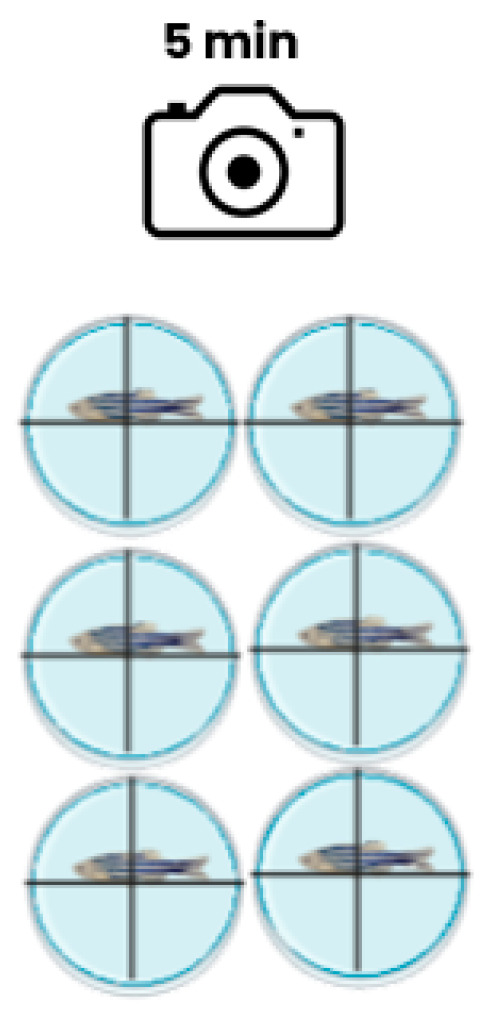
Representation of the open field test using the zebrafish animal model.

The open field test was performed to evaluate possible changes in motor coordination due to anxiolytic effects and/or muscle relaxation [32]. Animals (*n* = 6/group) were pretreated according to the general protocol. Diazepam (DZP; 4 mg/kg) and 3% DMSO were used as positive and negative controls, respectively. After 30 min, animals were individually placed in glass Petri dishes (10 × 15 cm) with grid markings on the bottom, containing aquarium water. The number of line crossings was recorded over a 0–5 min period (Figure 10).

#### 4.6.3. Anxiolytic Assessment

**Figure 11:**
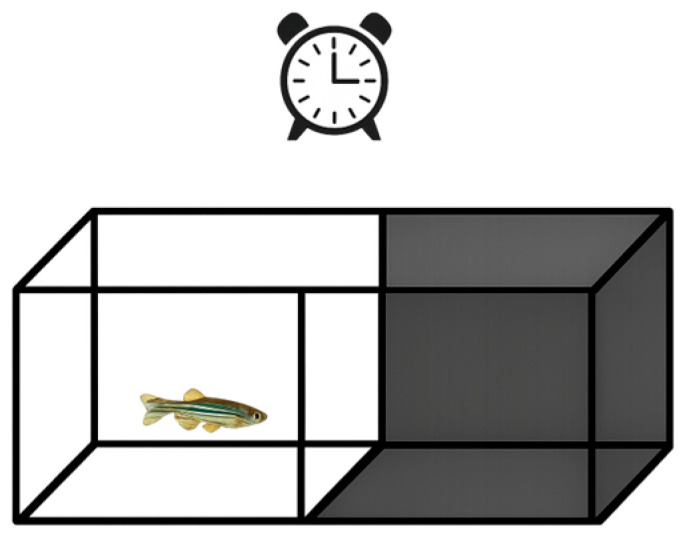
Representation of the light/dark test using the zebrafish animal model.

Anxiety-like behavior was evaluated using the light/dark test. Similar to rodents, zebrafish naturally avoid illuminated areas [33]. The experiment was conducted in a glass aquarium (30 × 15 × 20 cm) divided into a light and dark compartment (Figure 11). The aquarium was filled to a depth of 3 cm with dechlorinated tap water, simulating a novel shallow environment capable of inducing anxiety-like behavior. Animals (*n* = 6/group) were treated according to the general protocol. Negative and positive control groups received 3% DMSO and diazepam (4 mg/kg), respectively. After 30 min, fish were individually placed in the light zone, and anxiolytic activity was measured based on the time spent in the light compartment during a 5 min observation period [10].

#### 4.6.4. GABAergic Neuromodulation Assessment

GABAergic involvement in the anxiolytic effect was assessed by pretreatment with flumazenil (GABAA receptor antagonist) prior to the light/dark test [34]. Fish (*n* = 6/group) were pretreated with flumazenil (4 mg/kg; 20 µL; i.p.). After 15 min, the most effective anxiolytic dose identified in previous tests was administered. DMSO 3% (vehicle; 20 µL; i.p.) was used as the negative control. After 30 min, animals were subjected to the light/dark test.

#### 4.6.5. Serotonergic Neuromodulation Assessment

Animals (*n* = 6/group) were pretreated with cyproheptadine (5-HT2A antagonist), pizotifen (5-HT1 and 5-HT2A/2C antagonist), administered orally or intraperitoneally at 32 mg/kg, and granisetron (5-HT3A/3B antagonist, 20 mg/kg, oral route) [25]. After 30 min, samples under evaluation, fluoxetine (0.05 mg/kg; 20 µL; i.p.), 3% DMSO (20 µL; i.p.), and the most effective anxiolytic dose of the essential oil were administered. Subsequently, animal behavior was assessed using the light/dark test (5 min).

#### 4.6.6. Statistical Analysis

Results were expressed as mean ± standard error of the mean (SEM) for each group of six animals. After confirming data normality and homogeneity, differences among groups were analyzed by one-way analysis of variance (ANOVA), followed by Tukey’s post hoc test. All analyses were performed using GraphPad Prism software version 8.0.1. Statistical significance was set at 5% (*p* < 0.05).

### 4.7. Chemometric Analysis

Chemometric analysis was conducted using Principal Component Analysis (PCA), Hierarchical Cluster Analysis (HCA), and Weighted Theoretical Contribution calculations, implemented in RStudio using appropriate statistical packages, RStudio (Posit RStudio IDE) available in March/2026 version 2026.01.2+418. Initially, data related to the chemical composition of the essential oil (percentage of each compound and their physicochemical properties, such as XLogP3) were organized into the predictor variable matrix (X matrix), while bioactivity data related to serotonergic receptors (5-HT1, 5-HT2, 5-HT3) and open field (OF) behavior were organized as dependent variables (Y matrix). Thus, the overall bioactivity observed for the essential oil was equally replicated for each compound. Although this approach does not allow causal inference between individual compounds and biological effects, it enables exploration of multivariate trends and generation of initial hypotheses regarding compounds most associated with specific bioactivity profiles.

PCA was applied to evaluate compound distribution in multivariate space and identify clustering patterns based on chemical characteristics. HCA was performed using the X matrix to classify compounds according to chemical similarity, with results presented as dendrograms and heatmaps to facilitate visual interpretation of relationships between oil constituents and their potential contribution to observed activity.

To estimate the weighted contribution of chemical compounds to the overall bioactivity of the essential oil, a Weighted Theoretical Contribution calculation was performed. The percentage of each compound was normalized to obtain its relative fraction within the oil. For each evaluated bioactivity, the theoretical contribution of each compound was calculated by multiplying its relative fraction by the global bioactivity value, according to the equation below:

$$C_{ij}=\left(\frac{P_{i}}{\sum P}\right)\times Y_{j}$$

where:

Cij = theoretical contribution of compound i to bioactivity j
Pi = percentage of compound i in the oil
∑P = total sum of percentages of all compounds
Yj = observed global bioactivity value for variable j

This analysis theoretically and exploratorily evaluates the relative contribution of each identified compound and its lipophilicity (X matrix—predictor variables) to the global biological effects observed in bioassays involving serotonergic receptor interactions (5-HT1, 5-HT2, 5-HT3) and the open field behavioral test (OF) (Y matrix—dependent variables).

Intestinal absorption and central nervous system (CNS) penetration properties of the compounds present in the essential oil were predicted based on their chemical structures using the BOILED-Egg graphical model implemented in the SwissADME platform available at URL: http://www.swissadme.ch (Accessed 18 March 2026). Compounds located in the white region (egg white) indicate high gastrointestinal absorption, whereas compounds located in the yellow region (yolk) indicate high blood–brain barrier penetration.

## 5. Conclusions

The essential oil of *M. flexuosa* (EOMF) exhibited a favorable acute safety in adult zebrafish and produced a significant anxiolytic-like effect at non-toxic doses. Behavioral data demonstrated increased light preference in the light/dark paradigm, with a moderate reduction in locomotor activity insufficient to explain the effect solely through sedation. Pharmacological antagonism assays indicated that the anxiolytic response was independent of GABA-A receptor modulation at the benzodiazepine site and suggest that the mechanism of action predominantly involves serotonergic pathways, particularly the 5-HT1 and 5-HT2A/2C receptors.

Chemometric analyses revealed a possible statistical association between major isothiocyanates, particularly butyl and isobutyl isothiocyanates, and behavioral endpoints, which appear to be influenced by their relative abundance and physicochemical properties. However, due to the exploratory nature of the multivariate modeling, direct molecular interactions and compound-specific contributions require further mechanistic studies using isolated compounds to be experimentally confirmed.

Overall, the integration of phytochemical characterization, behavioral pharmacology, neuromodulatory assays, and silico predictions supports the neuroactive potential of EOMF and identifies serotonergic modulation as a central mechanistic pathway. These findings expand the pharmacological knowledge of *M. flexuosa* and support further investigation of isothiocyanate-rich essential oils as candidates for multi-target anxiety modulation strategies.

## Figures and Tables

**Table 1:** Chemical constituents of the essential oil from *Morisonia flexuosa* seeds.

Compounds		%	RT ^1^	RI ^2^
1	Butyl isothiocyanate	42.60	3.633	1.029
2	Isobutyl isothiocyanate	42.28	3.855	1.056
3	2-Methylbutyl isothiocyanate	1.02	5.081	1.160
4	Pentyl isothiocyanate	1.90	5.488	1.189
5	5-Butylnonane	0.44	7.388	1.290
6	2,6,10-Trimethyltetradecane	1.60	9.309	1.447
7	(−)-β-Elemene	1.28	9.386	1.456
8	*trans*-Caryophyllene	1.53	10.362	1.553
9	Dotriacontane	1.52	13.865	1.786

**RT ^1^**: Retention time (min); **RI ^2^**: Experimental Kovats relative retention indices. *n*-Alkanes (C8–C40) were used as reference points for the calculation of relative retention indices.

## Data Availability

The original contributions presented in this study are included in the article. Further inquiries can be directed to the corresponding author.

## Abbreviations

The following abbreviations are used in this manuscript:

GSLs	Glucosinolates
ITCs	Isothiocyanates
EOMF	Essential Oil *Morisonia flexuosa*
CG	Gas Chromatography
FID	Flame Ionization Detector
CNS	Central Nervous System
PCA	Principal Component Analysis
PC	Principal Component
CB2	Cannabinoid Receptor Subtype 2
5HT	5-hydroxytryptamine
5HT (1, 2, 3)	Serotonergic Receptors
OF	Open Field
LogP	Octanol-Water Partition Coefficient
5-HT2A	Serotonin Subtype 2A Receptor
5-HT2C	Serotonin Subtype 2C Receptor
HCA	Hierarchical Cluster Analysis
